# Predictive computational modeling to define effective treatment strategies for bone metastatic prostate cancer

**DOI:** 10.1038/srep29384

**Published:** 2016-07-14

**Authors:** Leah M. Cook, Arturo Araujo, Julio M. Pow-Sang, Mikalai M. Budzevich, David Basanta, Conor C. Lynch

**Affiliations:** 1 Tumor Biology Dept, Moffitt Cancer Center and Research Institute, Tampa, Florida, USA; 2 Integrated Mathematical Oncology Dept, Moffitt Cancer Center and Research Institute, Tampa, Florida, USA; 3 Genitourinary Oncology Dept, Moffitt Cancer Center and Research Institute, Tampa, Florida, USA; 4 Dept. Cancer Imaging and Metabolism, Moffitt Cancer Center and Research Institute, Tampa, Florida, USA

## Abstract

The ability to rapidly assess the efficacy of therapeutic strategies for incurable bone metastatic prostate cancer is an urgent need. Pre-clinical *in vivo* models are limited in their ability to define the temporal effects of therapies on simultaneous multicellular interactions in the cancer-bone microenvironment. Integrating biological and computational modeling approaches can overcome this limitation. Here, we generated a biologically driven discrete hybrid cellular automaton (HCA) model of bone metastatic prostate cancer to identify the optimal therapeutic window for putative targeted therapies. As proof of principle, we focused on TGFβ because of its known pleiotropic cellular effects. HCA simulations predict an optimal effect for TGFβ inhibition in a pre-metastatic setting with quantitative outputs indicating a significant impact on prostate cancer cell viability, osteoclast formation and osteoblast differentiation. *In silico* predictions were validated *in vivo* with models of bone metastatic prostate cancer (PAIII and C4-2B). Analysis of human bone metastatic prostate cancer specimens reveals heterogeneous cancer cell use of TGFβ. Patient specific information was seeded into the HCA model to predict the effect of TGFβ inhibitor treatment on disease evolution. Collectively, we demonstrate how an integrated computational/biological approach can rapidly optimize the efficacy of potential targeted therapies on bone metastatic prostate cancer.

Metastatic castrate resistant prostate cancer (mCRPC) typically manifests in the skeleton and is currently incurable^1^,^2^. In the bone microenvironment, prostate cancer cells hijack the normal bone remodeling process to create a “vicious cycle” of extensive bone formation and destruction^3^. Key mechanisms facilitating the cross-talk between the cancer and host compartment include the induction of receptor activator of nuclear κB ligand (RANKL) expression and the release of sequestered growth factors from the bone matrix. Bone is a rich source of transforming growth factorβ (TGFβ) and the role for this pleiotropic factor in promoting the survival and growth of bone metastatic cancers has been well described^4^,^5^. The molecular complexity of the circuitry driving this cycle has expanded tremendously in the past two decades revealing many potential targets for therapeutic intervention. The question remains however as to how to translate these potential therapies to the clinic. Biological experimentation and pre-clinical mouse models can be used to define the impact of putative therapies but are limited in their ability to dissect the potential dynamic and simultaneous effects on the multi-cellular tumor-bone microenvironment. One potential alternative approach is the integration of experimentally measured biological parameters with computational models to tackle the multi-scale nature of the disease^6^. Numerous computational models successfully demonstrate the feasibility of the approach^7^,^8^,^9^,^10^,^11^,^12^,^13^,^14^. Starting from existing experimental or clinical data it is possible to use statistical frameworks such as Approximate Bayesian Computation (ABC) to identify, in a “top-down” manner, the importance of unknown parameters in disease progression by applying a distribution of probability on those factors^15^. Conversely, agent based models, such as discrete-continuum Hybrid Cellular Automata (HCA), are better suited to test hypotheses using a mechanistic “bottom-up” approach to provide unbiased predictions^16^. These models work by parameterizing the properties of cells (or agents) with regards to proliferation, apoptosis, secretion of factors, genetic mutations or even metabolism^17^. The ability to apply HCA models to two- or three-dimensional grids make them uniquely qualified for studying temporal tumor-host interactions over time, especially in the context of applied therapies^15^,^18^,^19^,^20^.

Previously, our group generated a HCA based computational model of the bone modeling unit (BMU) that recapitulates the homeostatic sequence of bone resorption and anabolism^18^. The *in silico* BMU is 1000 μm × 1500 μm and is composed of bone, mesenchymal stromal cells (MSCs), precursor and adult osteoblasts, and precursor and mature multinucleated osteoclasts. The sequence and timing of resorption and bone formation that emerges from the model recapitulates the extensive literature and the interactions of the cells were carefully modeled around bone derived factors including RANKL and TGFβ^18^. Using human parameters based on the growth of prostate cancer in bone we demonstrated that the introduction of an emboli of prostate cancer cells (≥9) into the BMU was sufficient to consistently initiate the vicious cycle. Subsequently, cancer-bone interaction could be monitored over a clinically relevant 250-day period^21^. We also reported how the model could be used to potentially optimize the effects of bisphosphonates and anti-RANKL therapies that are components of the current standard of care. In the current study, a major objective was to use the model to explore the impact/efficacy of putative inhibitors. Our previously published HCA model, as expected, defined an important role for TGFβ in regulating cancer-bone interaction^18^.

TGFβ inhibitors such as neutralizing antibodies are currently undergoing clinical trial^22^. However, their application for the treatment of osteogenic bone metastatic prostate cancer has not been explored thus far due to the pleiotropic and often opposing effects TGFβ can have on cancer and bone cell behavior^5^,^23^,^24^,^25^. Therefore, we posit that TGFβ inhibition would be an ideal challenge for testing the predictive power of our HCA based model. Here, using an evolved version of the HCA model, we simulated various therapeutic strategies (i.e. inhibitor concentration, time of therapeutic intervention) to predict the optimal efficacy of TGFβ inhibition. Further, the enhanced HCA model provides new insights into how TGFβ can regulate multi-cellular interactions over time. HCA outputs were validated *in vivo* using two models of osteogenic bone metastatic prostate cancer. Moreover, using patient specific information from bone metastatic specimens, we demonstrate the flexibility of the HCA model in predicting the efficacy of TGFβ inhibitors on lesions that are heterogeneous for TGFβ utilization. Collectively, we demonstrate how an integrated computational/biological modeling approach can be used to optimize therapy efficacy for the treatment of bone metastatic prostate cancer.

## Results

### Computational modeling of TGFβ inhibition in normal bone remodeling and in bone metastatic prostate cancer

**Figure 1:**
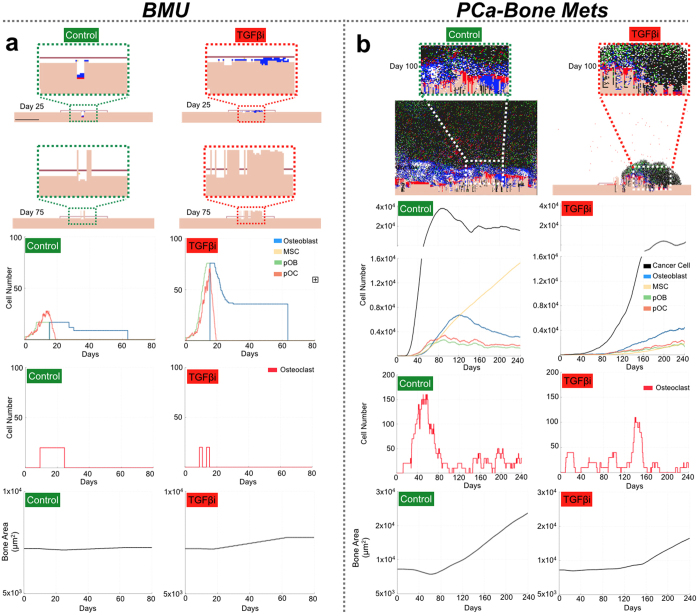
*In silico* effects of TGFβ inhibition on normal and prostate cancer induced bone turnover (**a**,**b**) *In silico* control and TGFβ inhibitor treated simulations in normal (BMU, n = 29/group, **a**) and bone metastatic prostate cancer (PCA-Bone Mets, n = 24/group, **b**) scenarios. Representative images of simulation runs at indicated time points are shown with magnified insets. TGFβ inhibitor was applied at day 1 for all simulations (pre-treatment scenario). Cell populations analyzed include mesenchymal stem cell (MSC), osteoblast precursors (pOB), osteoblasts, osteoclast precursors (pOC) and osteoclasts. Temporal changes in bone area (μm^2^) were also predicted under control and TGFβ inhibitor conditions.

TGFβ is known to have concentration dependent pleiotropic effects on osteoblasts and osteoclasts^26^,^27^,^28^. *In silico*, the ability of stromal cells to respond to varying TGFβ concentrations (0.1 to 10 ng/ml) was integrated into our HCA of normal bone remodeling, the bone multicellular unit (BMU) (see Equations 1, 2, and Table S1)^18^. To obtain a realistic readout of the level of TGFβ inhibition that could be achievable *in vivo*, we treated mice with a TGFβ neutralizing antibody (1D11) at a dose previously used in the literature (10 mg/kg) and consistent with clinical trials performed for a humanized version of the 1D11 TGFβ neutralizing antibody, fresolimumab/GC1008^22^,^29^,^30^. We observed that the TGFβ neutralizing antibody significantly reduced circulating TGFβ serum levels by up to 80% compared to IgG control treated animals (Supplementary Fig. S1). Using phospho-SMAD2 as a surrogate for TGFβ activity^31^, we also observed that the TGFβ neutralizing antibody could inhibit TGFβ activity 50–80% in tumor naïve and tumor bearing tissues (Supplementary Fig. S1 and Fig. 3f). Based on this *in vivo* information, we applied the TGFβ inhibitor to the normal BMU at a level of 80% *in silico*. The stochastic nature of the BMU allows for variation and statistical analysis of simulation outputs. The results of multiple simulations (n = 29/group) show that TGFβ inhibition significantly promoted bone formation (9% increase) over a 75-day period by enhancing osteoblast expansion and differentiation while limiting osteoclast viability (Fig. 1a, Supplementary Fig. S1, and Table S2). Importantly, these *in silico* results are consistent with previous *in vivo* studies and support the robustness of the parameters used to power the computational model^29^,^32^.

The vicious cycle paradigm suggests that metastatic prostate cancer cells utilize TGFβ signaling to promote their survival and growth. We therefore seeded the computational model with TGFβ responsive prostate cancer cells. Once the vicious cycle was established at day 80, we initiated TGFβ inhibition (post-treatment scenario). Simulations (n = 24/group) revealed that TGFβ inhibition reduced cancer growth by approximately 15%, but only when the inhibitor was applied at a constant 99% level of efficacy until day 250 (Supplementary Fig. S2 and Video 1). At a more biologically relevant level of 80% inhibition, we observed little difference in cancer cell growth between the control and treatment groups. Surprisingly, and in contrast to the observed effects of TGFβ inhibition on the normal BMU, we also observed no difference in osteogenesis between the control and treatment groups even at later stages (Supplementary Fig. S2, Table S3, and Video 1). Taken together, these results suggest that the treatment of established and actively growing bone metastases with TGFβ inhibitors would have no impact on the progression of the lesions unless the inhibitor was >99% effective.

A major advantage of the computational model is that it can be used to explore therapeutic windows of efficacy for putative inhibitors. Simulations (n = 24) revealed that applying the TGFβ inhibitor *in silico* at day 1 prior to the seeding of the cancer cells (pre-treatment scenario) even at a level of 20% efficacy significantly reduced tumor burden over time by ≥65% (Fig 1b, Supplementary Fig. S2, Table S3, and Video 2). Interestingly, TGFβ inhibition resulted in a small but significant increase in cancer-induced osteogenesis compared to control during early tumor progression (Day 100). However, at the end of the simulations there was significantly less cancer-induced osteogenesis in the TGFβ treated group compared to control (Fig. 1b, Supplementary Fig. S2, and Table S3, Day 250). These *in silico* results predict that applying TGFβ inhibitors in a preventative manner will reduce the growth of metastatic prostate cancer without exacerbating cancer induced osteogenesis.

### *In vivo* validation of computational model predictions

**Figure 2:**
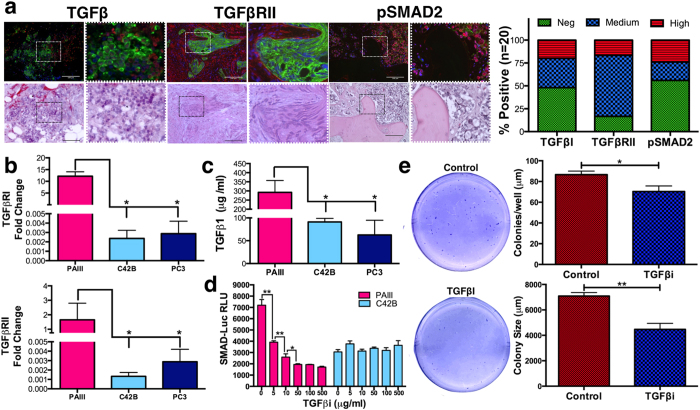
TGFβ expression and utilization in prostate cancer specimens and cell lines. (**a**) Immunofluorescence of TGFβ, TβRII, and pSMAD2 (red) in human (cytokeratin-green) bone metastatic prostate cancer (n = 20). Dashed box represents area of magnification. Graphs represent intensity of pixels. Scale bars represent 100 μm. (**b**,**c**) Real time PCR analysis of TβRI and TβRII expression (**b**) and ELISA measurement of TGFβ concentration (**c**) in PAIII, C42B and PC3. (**d**) The effect of increasing concentrations of TGFβ inhibitor (TGFβi; 1D11 antibody) on SMAD reporter activity (RLU). (**e**) The effect of TGFβ inhibition (TGFβi; 1D11 10 μg/ml) on colony formation and size compared to control (Control-13C4, 10 μg/ml). Asterisks denote statistical significance (*p < 0.05; **p < 0.01).

Analysis of human specimens of bone metastatic prostate cancer derived from deidentified cancer patients at the Moffitt Cancer Center (n = 20) show that TGFβ ligand and receptors are expressed and pSMAD2 staining indicates that the TGFβ pathway is active (Fig. 2a). *In vitro* analysis of prostate cancer cell lines that can grow in the bone microenvironment identified the PAIII cell line as reflecting the TGFβ receptor and growth factor producing (TRP) status observed in human specimens (Fig. 2b,c). We also noted that the PAIII cell line was sensitive to inhibition with TGFβ inhibitors (Fig. 2d,e). We therefore initially chose this cell line to test computational model predictions.

**Figure 3:**
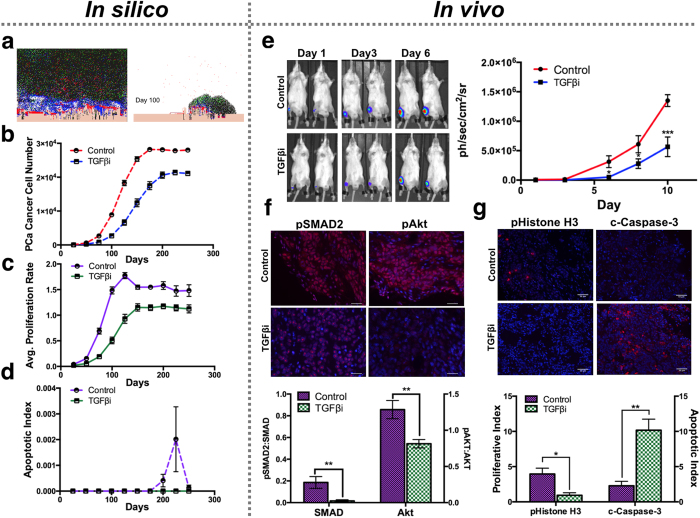
TGFβi pre-treatment prevents bone metastatic prostate cancer growth *in silico* and *in vivo*. (**a**) Representative *in silico* outputs from control (left panel) and TGFβi (right panel) treated simulations at day 100. (**b**–**d**) *In silico* predictions of TGFβ inhibition on cancer cell growth (**b**), and cancer cell proliferative/apoptotic rates (average number of proliferating/apoptotic cancer cells at 25 day intervals over a 250 day period, (**c**,**d**). (**e**) Bioluminescence measurement of PAIII growth under TGFβi (1D11, 10 μg/ml; n = 10) or control (13C4, 10 μg/ml; n = 8) conditions. (**f**) pSMAD2 and pAKT positivity (red) as a ratio of unphosphorylated protein. Scale bars, 25 μm. (**g**) The proliferative and apoptotic index in TGFβi and control tissue sections were measured using pHistone H3 and cleaved caspase-3 (c-Caspase-3) (red) respectively as a ratio to total cell number (DAPI; blue). Scale bars, 50 μm. Asterisks denote statistical significance (*p < 0.05; **p < 0.005).

Dissecting cancer cell behavior *in silico* illustrates TGFβ inhibition directly limits growth over time by impacting cancer cell proliferation (Fig. 3a–d). To determine the validity of the computational outputs, 6-week-old male SCID Beige mice were pre-treated with either a TGFβ inhibitor (TGFβi-1D11, 10 mg/Kg, 3× weekly; n = 10) or an isotype control IgG (Control-13C4, 10 mg/Kg 3× weekly; n = 8) and subsequently inoculated with luciferase-expressing PAIII cells. Bioluminescence analysis revealed a significant reduction in tumor growth in the TGFβ inhibitor treated group compared to controls and, as expected, reduced pSMAD2 and AKT phosphorylation (Fig. 3e,f). We further found significant reductions in proliferation (40%) and increases in apoptosis (70%) between the TGFβ inhibitor treated and control groups (Fig. 3g). The HCA model is based on humanized parameters and therefore, to compare computational model outputs to those obtained with the PAIII model, we used a scaling approach. Briefly, using tumor growth rates from the computational model (25 day intervals) and the animal model (2 day intervals) we calculated the slope of the line/derivative for each time point. Using this approach, we found that derivatives for each model are similar for pre and post treatment between day 8–10 for the animal model and day 75–100 for the *in silico* model. At these comparable points, the computational model accurately predicts the impact of TGFβ inhibition on cancer cell proliferation but differences in apoptosis were not evident in the computational model until later time points (Fig. 3d vs. 3g).

To further assess the predictive power of the computational model, we performed a TGFβ inhibitor post-treatment study. *In silico* findings suggest treatment of an established TGFβ ligand and receptor-expressing cancer (TRP) with a TGFβ inhibitor would not be of benefit. Our *in vivo* data confirm these predictions (Supplementary Fig. S3).

### TGFβ inhibition prevents prostate cancer induced osteolysis

**Figure 4:**
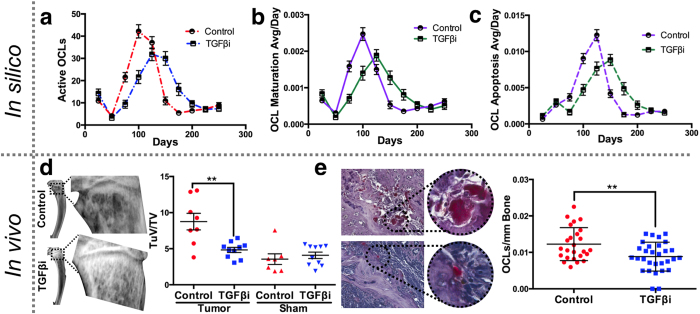
TGFβi effects on cancer induced osteolysis. (**a**) Osteoclast activity in the TGFβi and control groups was determined *in silico* at 25 day intervals over a 250 day period. (**b**,**c**) The effect of TGFβi versus control on osteoclast fusion/maturation and apoptosis over time as a ratio to total cell number. (**d**) Ratio of tumor volume (measured by total area of osteolysis; TuV) to total volume (TV) in X-rays of tumor and sham tibia from TGFβi (n = 10) and control (n = 8) groups. (**e**) Quantitation of TRAcP (red) positive osteoclasts per tumor/bone interface. Asterisks denote statistical significance (*p < 0.05; **p < 0.005; ***p < 0.0001).

Because of their role in the vicious cycle, we next focused on osteoclast behavior over time in the control and TGFβ inhibitor treated groups. Dissection of the computational model outputs revealed the number of active bone resorbing osteoclasts were significantly lower in the TGFβ inhibitor versus control group at day 100 (Fig. 4a). Further, the rate of osteoclast maturation and apoptosis was significantly mitigated during this period, which is in agreement with known effects of TGFβ inhibition on osteoclast function (Fig. 4b,c)^28^,^33^. Analysis of the lytic component of our *in vivo* model identified that there was approximately 50% (p = 0.002) less tumor induced osteolysis in the TGFβ inhibitor group compared to the controls as determined by X-ray (Fig. 4d). Histochemical analysis using the osteoclast specific marker tartrate resistant acid phosphatase (TRAP) demonstrated that this reduction in osteolysis was due to significantly fewer osteoclasts in the TGFβ inhibitor treated group compared to the controls (Fig. 4e). These data suggest TGFβ inhibition reduces the extent of cancer-induced osteolysis over time *in vivo* by limiting osteoclast function and validates the pre-treatment HCA model outputs.

### TGFβ inhibition has differential effects on normal and cancer induced bone formation

**Figure 5:**
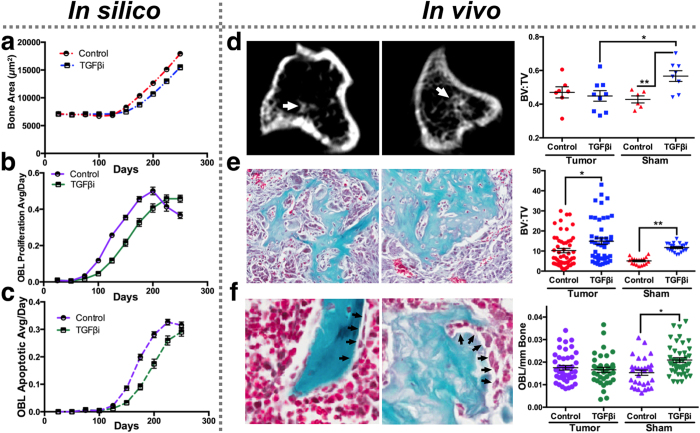
TGFβi impact on osteogenesis. (**a**) *In silico* changes in bone area (μm^2^) were predicted at 25-day intervals over a 250 day period. (**b**,**c**) The effect of TGFβ inhibition on the average number of proliferative and apoptotic osteoblasts per day *in silico*. (**d**) μCT analysis of bone volume (arrow) to total volume (BV:TV) in control and TGFβ inhibitor treated bones. (**e**,**f**) Histomorphometry analysis of osteogenesis (**e**) and number of bone rimming osteoblasts (arrows; **f**). Asterisks denote statistical significance (*p < 0.05; **p < 0.005; ***p < 0.0001).

Based on published data demonstrating that TGFβ blockade increases bone formation^29^, we hypothesized that inhibition of the growth factor would significantly exacerbate prostate cancer induced osteogenesis. At day 100, the computational model does predict a small but significant increase in bone formation in the TGFβ inhibitor simulations despite a concomitant decrease in osteoblast proliferation (Fig. 5a–c and Supplementary Fig. S2). While microCT (μCT) scans of tumor bearing bones did not reveal any differences between the groups (Fig. 5d), histomorphometry analysis does support the *in silico* predictions with increased bone formation in TGFβ inhibitor treated mice and a trend towards fewer bone rimming osteoblasts (Fig. 5e,f). In contrast, we observed a robust increase in bone formation in contralateral sham limbs derived from the TGFβ inhibitor group for all measurements (Fig. 5d–f). Taken together, these *in vivo* data suggest that TGFβ inhibition does not greatly exacerbate prostate cancer induced osteogenesis compared to non pathological conditions and support, in part, HCA model outputs. It is also worth noting that over a longer period (Day 250), the computational model predicts TGFβ inhibition would ultimately result in decreased bone formation (Fig. 5a and Supplementary Fig. S2), a finding that warrants further exploration *in vivo*.

### Predicting TGFβ inhibitor efficacy on heterogeneous bone metastatic prostate cancer

While the majority of cancer cells in human specimens of bone metastatic prostate cancer produce both TGFβ ligand and receptors (TRP), we also noted the presence of cancer cells that either produced the ligand alone (TP), the receptor alone (TR) or neither (TN) (Fig. 2). This raises the question as to what the impact of TGFβ inhibition would be on these clonal populations. We have shown that, in our hands, the C4-2B cell line produces TGFβ but not the receptors (TP) (Fig. 2b–d). Therefore, we challenged the computational model to determine the impact of TGFβ inhibition on a homogenous TP bone metastatic cancer and found no effect on the growth of the cancer in pre- or-post treatment simulations (Equation 5, Supplementary Fig. S4). To test these results *in vivo*, C4-2B luciferase-expressing cells were inoculated into mice that were pre- or post-treated with TGFβ inhibitor or IgG control and we observed no difference in tumor growth between the groups (Supplementary Fig. S4).

**Figure 6:**
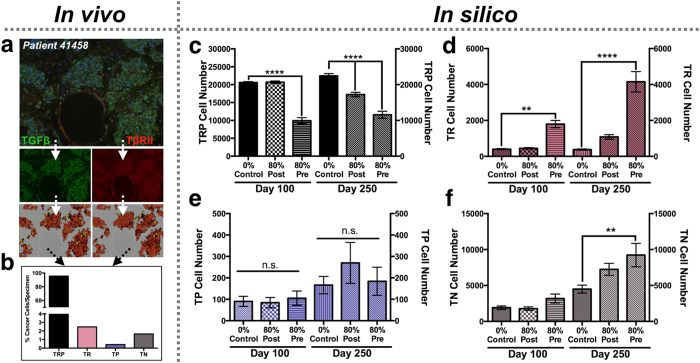
*In silico* effects of TGFβ inhibition on heterogeneous bone metastatic prostate cancer. (**a**) Individual human specimen of bone metastatic prostate cancer (Patient 41458) co-stained for TGFβ (green) and TβRII (red). (**b**) Graph of staining pixel intensity for each clonal population. (**c**–**f**) *In silico* simulations (n ≥ 24/group) were performed under control or TGFβ inhibition (80% efficacy) post- and pre-treatment conditions. Clonal population was measured at Day 100 (left y-axis) and Day 250 (right y-axis). Asterisks denote statistical significance (**p < 0.005; ****p < 0.0001).

Clinically, human samples of bone metastatic prostate cancer are heterogeneous for their usage of TGFβ (Fig. 2a). A major advantage of the HCA modeling approach is the ability to integrate multiple clonal phenotypes based on patient specific information. In this regard, analysis of a single patient specimen, identified heterogeneous expression and activity in the TGFβ signaling axis with the following clonal ratio noted: TP 1: TRP 231: TR 6: TN 4 (Fig. 6a,b). We seeded the HCA model with this ratio of clones and performed simulations for control, pre- and post-treatment conditions (n ≥ 24/group) to determine the impact of TGFβ inhibition on metastatic prostate cancer evolution (Equations 3, 4, 5, 6). *In silico* data show the effects of pre- and post- TGFβ inhibitor treatment on clonal evolution for this patient over time and the responses of the surrounding tumor microenvironment (Supplementary Fig. S5, Table S4, and Video 3). The model also predicts the dominance of the TRP clone over time under control conditions (Fig. 6c). However, application of the TGFβ inhibitor, especially in the pre-treatment groups at varying levels of efficacy, show how the cancer cell dynamics shift over time in favor of other clones, in particular for the TN population (Fig. 6d–f and Table S4). These data underscore the power of the HCA computational modeling approach in examining the temporal effects of targeted therapy on heterogeneous cancer cells and the surrounding microenvironment.

## Discussion

Current treatment options for patients diagnosed with bone metastatic castrate resistant prostate cancer include second generation androgen deprivation therapies, radiation treatment, bisphosphonates/anti-RANKL therapies, alpharadin and/or chemotherapy^1^. While these treatments mitigate pain, pathological fracture and increase overall survival, the disease remains incurable with the median survival time being approximately 3-years subsequent to diagnosis. Increasing our knowledge of the mechanisms driving the disease can reveal novel therapeutic targets. To this end, dozens of molecular mechanisms that play important roles in tumor-bone interaction have been discovered^34^. However, dissecting how potential targeted therapies will work in the context of current treatment paradigms and their translation to the clinical setting presents a major challenge. Using TGFβ inhibition as an example, we demonstrated how a novel biologically driven computational HCA model can rapidly define temporal cancer-bone microenvironment responses to a given therapy. Further, the integrated approach provides insight into optimal therapeutic windows to apply a given inhibitor. For TGFβ inhibition, the HCA model indicates that application of the inhibitor in an adjuvant setting subsequent to the detection/treatment of aggressive prostate cancer would be most effective.

As with biological models, there are numerous mathematical approaches to study cancer progression such as branching and Moran processes based models, systems of ordinary and partial differential equations, and agent based models, each with varying strengths and weaknesses^6^,^15^,^35^,^36^. Data driven “top down” models “fit” existing clinical or experimental information to identify parameters that explain the behavior of the disease^37^,^38^. In the context of prostate cancer, many elegant models have used biological parameters such as prostate specific antigen (PSA) to predict time to progression, and to model the effects of intermittent androgen therapy^8^. The tightly regulated process of normal bone remodeling lends itself well to modeling how key factors such as RANKL and TGFβ control the behavior and activity of bone stromal cells over time^39^,^40^,^41^,^42^,^43^. By extension, perturbing this balanced ecosystem with an invasive species such as cancer can also be modeled. Our own group, as well as others, has been exploring how bone metastatic cells and skeletal malignancies such as myeloma interact with the bone microenvironment in order to progress^18^,^44^,^45^. In the current study, we used an agent-based approach that allows for the exploration of key cellular interactions over space and time in an unbiased hypothesis driven “bottom-up” manner. We have shown how experimentally-derived cellular parameters can be integrated into the rule sets and partial differential equations (PDEs) used to drive HCA models. This allows us to produce biologically^46^ and clinically^47^ testable hypotheses without making assumptions regarding population level dynamics. The result is a model capable of generating predictions that naturally emerge from the interactions between cells and their environment. Key to the robustness of the HCA model outputs, is the reliability of the biological parameters used to power the PDEs. In the current study, we used human parameters to generate the HCA model^21^. While we assume that individual bone stromal cell components have similar dimensions and lifespans between the species, human cancer cells typically grow much more slowly than human derived xenografts or animal models of the disease^21^. Therefore appropriate scaling between the models is an important consideration for the comparison of results. Further, not all of the HCA models predictions were correct. For example, we observed the *in silico* effects of TGFβ inhibition on osteoblast number was discordant with *in vivo* results (Fig. 5b vs. 5f), suggesting that the parameters/assumptions governing the effects of TGFβ inhibition on apoptosis require re-calibration based on the obtained biological data. This reiterative process allows for fine-tuning of the HCA.

The roles of TGFβ in skeletal development and malignancy have been well described^5^,^24^. However, enthusiasm for applying TGFβ inhibitors as a therapeutic strategy to treat metastatic bone disease including mCRPC is limited because of the pleiotropic and often opposing roles TGFβ plays in normal and cancer cell biology^5^,^24^,^25^,^48^,^49^. This complexity made TGFβ and the effects of TGFβ inhibition an ideal challenge with which to test the power of our HCA based computational model. Our results indicate the treatment of established active metastases with a TGFβ inhibitor, unless applied at >99% efficacy, would have little or no impact on the progression of cancer cells regardless of their dependency on TGFβ. In contrast, the application of the inhibitor in a preventative or adjuvant manner would significantly control bone metastatic prostate cancer growth and osteogenesis. This result was predicated on the basis that the metastatic cancer cells have an active TGFβ signaling axis which we found to be the case in the majority of human bone metastatic prostate cancer specimens. The mathematical model was built on the assumption that TGFβ, RANKL and bone derived nutrients drive cell responses. Arguably then, interfering with TGFβ signaling in the model could potentially be self-fulfilling in predicting cancer-host behavior. However, despite the limited number of cytokines and growth factors included in the model, we validated many of the model predictions with independent *in vivo* experiments thus reinforcing the key roles for TGFβ and RANKL in the vicious cycle of tumor bone interaction. The majority of our *in vivo* results support the accuracy of the parameters and assumptions used to power the computational model. The *in vivo* results are consistent with other studies examining the role of TGFβ in bone metastatic cancers including prostate, breast and melanoma but, importantly, underscores the potential for computational modeling in predicting the efficacy of an applied targeted therapy^50^,^51^,^52^. Although our model is centered on the roles of TGFβ and RANKL, it is important to note that the circuitry of the HCA model can easily be expanded to include other cell types (immune cells or cancer-associated fibroblasts) and molecules (e.g. PTHrP, BMPs) and their roles/effects subsequently explored.

While the computational model was primarily used to examine TGFβ inhibition in bone metastatic prostate cancer, the outputs generated also revealed new insights into TGFβ biology. Firstly, the model highlights the critical role for TGFβ in the cyclical dynamics of populations such as osteoclasts and confirms the importance of the osteoclast in initializing the vicious cycle. For example, we noted osteoclast infiltration often precedes a period of cancer growth and osteogenesis (Video S2), a finding that could be informative for the timing of anti-resorptive therapies as we have previously shown^18^. TGFβ inhibition alters these dynamics and at specific time-points can reverse the trends between the treated and control groups so that osteoclast numbers in the control simulations may in fact be lower than those in the TGFβ inhibitor treated group (Fig. 3A). This suggests that arbitrarily selected time-points in pre-clinical *in vivo* animal studies may not accurately reflect how applied therapeutics are impacting cancer-bone interaction over time. Secondly, although TGFβ inhibition promotes robust bone formation in normal non-pathological situations, the model and our *in vivo* results confirm counter-intuitively that, TGFβ inhibition does not greatly exacerbate prostate cancer induced osteogenesis.

Currently, mCRPC inter-patient heterogeneity is a major clinical challenge. Integrating biological and computational modeling offers a unique opportunity to study how cancer evolves and reacts to changing microenvironments and applied targeted therapies. Patient derived xenograft (PDX) samples are being used to design precision treatment strategies and integrating the biological parameters derived from these specimens into computational models could prove to be a synergistic way to tackle the complexity of heterogeneity in individual patients^19^. Again, using patient derived TGFβ signaling axis information as an example, we demonstrated how clonal variation and evolution in response to applied TGFβ inhibitors could be incorporated into the HCA model (Fig. 6). In these studies, we assumed the TGFβ dependent growth rates of clonal cancer cells. For personalization of the HCA and its clinical application, individual patient specimens would have to be isolated and growth rates of various clones examined in *ex vivo* assays. Current advances in single cell *ex vivo* analyses support the feasibility of such an approach^53^,^54^. The HCA model also allows for the optimization of inhibitor dosing and timing that in turn could be used to generate an adaptive therapy strategy to prevent the outgrowth of resistant sub-populations^20^,^55^.

In conclusion, we have developed a novel and unbiased computational HCA model that allows for the dynamic multi-scale understanding of how metastatic prostate cancer cells evolve and interact with the surrounding bone microenvironment. We used this model to predict the efficacy and response of bone metastatic prostate cancer to targeted therapies such as, TGFβ inhibitors. Further, our integrated computational and biological approach allowed for the dissection of how TGFβ inhibition simultaneously affects osteoblast, osteoclast and cancer cell behavior over time. The HCA model constitutes a platform for discovery that can readily be expanded to incorporate additional cellular and molecular circuitry. This will ultimately yield a clinical tool that will aid the medical oncologist in designing curative strategies for heterogeneous bone metastatic prostate cancer. Most importantly, this modeling approach can be applied to the development of new therapeutic strategies across a broad spectrum of human malignancies.

## Methods

### An HCA Model of bone metastatic prostate cancer for therapy optimization

In the computational model, each cell type responds to TGFβ levels in an either directly proportional (1 + *Log*(*TGFβ*)) or inversely proportional (−1*Log*(*TGFβ*)) manner. TGFβ inhibition in the HCA is achieved by controlling TGFβ bioavailability (0–99% inhibition at a constant level). Computational models were seeded with homogeneous or heterogeneous prostate cancer cells that expressed the TGFβ receptor and ligand (TRP), the ligand alone (TP), the receptor alone (TR) or were negative for both (TN). For the HCA model, we consider cellular intrinsic behaviors and the impact of TGFβ on these behaviors. We include 8 different cell types: resident and active cells of the bone stroma (mesenchymal stromal cells (MSCs), precursor osteoblasts (pOB), adult osteoblasts (aOB), precursor osteoclasts (pOC), adult osteoclasts (aOC)) and prostate cancer cells of varying TGFβ responsiveness (TRP, TGFβ ligand and receptor-producing; TR, receptor-producing; TP, ligand-producing and; TN, negative for receptor and ligand expression). We considered interactions between all cell types and the impact of these interactions on the tumor-bone microenvironment. Empirical, experimental and theoretical parameters were used to fuel equations (Table S1).

#### Precursor Osteoblasts and Osteoclasts

Based on empirical data and literature, we assume a maximum rate of pOB division as 36 hours based on ATCC specifications for MC3T3-E1 cells. The rate of pOB division is inversely affected by TGFβ^28^ and we assume that the effect is logarithmic. If TGFβ is at saturation levels (>10 ng/ml), then the pOB division rate tends to zero. This assumption is based on our findings of the effect of TGFβ on osteoblast precursors (Supplemental Fig. S1). If there is no TGFβ present, then the maximum rate is considered. The rate of division has subsequent effects on the number of mature bone generating osteoblasts. By the same token, the fusion rate of pOC is also affected inversely proportional to the availability of TGFβ. These behaviors can be described by:


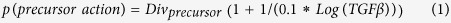


where 0 < TGFβ < 1 and *Div*_*precursor*_ is substituted in the case of precursor osteoblasts, *Div*_*pOB*_ is the maximum rate of pOB division, and in the case of precursor osteoclasts, *Div*_*pOC*_, the maximum rate of pOC fusion in the absence of TGFβ. This ensures that, when bone is being resorbed and TGFβ is being made bioavailable, osteoclastogenesis is limited.

Once fused, the probability of aOC survival also depends proportionally on TGFβ. This is calculated by:


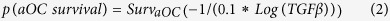


where 0 < TGFβ < 1 and *Surv*_*aOC*_ is the maximum percentage of survival for aOCs (100% when TGFβ is as its saturation level). If the levels of TGFβ are below the saturation level, the probability of death for the aOC increases^56^,^57^.

#### Bone metastatic prostate cancer cells

Prostate cancer cells were explicitly defined as being dependent on TGFβ and bone derived nutrients (*BDN*) for their division. The probability of division was estimated as being proportional to the inverse logarithm of the available BDN. If there are no nutrients, there is zero division. If there is maximum nutrient saturation, the division rate is at its maximum.

Based on empirical data obtained with TRP cells such as PAIII, we assume that TRP cells have a maximum division rate of once every 1.5 days and a lifespan without contact with BDN of 14 days based on low serum (2%) soft agar assays (Supplementary Fig. S4). TRP division in response to bone derived nutrients was modeled as follows:


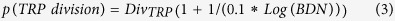


where 0 < TGFβ < 1 and *Div*_*TRP*_ is the maximum rate of TRP division, in the saturation level of bone derived nutrients. Under TGFβ inhibition, the maximum division rate is assumed to be reduced for TRP to once every 2 days based on empirical observations in our laboratory (data not shown).

We assume that TR has a maximum division rate of 1.75 days, but a lifespan without nutrients of 10 days, calculated as not having a cost for producing TGFβ ligand but benefiting from the presence TGFβ in the bone microenvironment. TR cell division depends directly on bone-derived nutrients in the same manner:


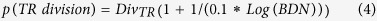


where 0 < BDN < 1 and *Div*_*TR*_ is the maximum rate of TR division, in the saturation level of bone derived nutrients. Under TGFβ inhibition, the maximum division rate is reduced for TR to once every two days.

We assume that TP has a maximum division rate of once 1.75 days, and a lifespan without nutrients of 10 days, calculated as having a cost for producing TGFβ ligand but not benefiting from TGFβ contained within the bone derived nutrients. TP cell division depends directly on bone-derived nutrients in the same manner:


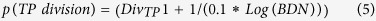


where 0 < BDN < 1 and *Div*_*TP*_ is the maximum rate of TP division. Under TGFβ inhibition, the maximum division rate of TP remains the same.

Finally, we assume TN has a maximum division rate of once every 2 days and a lifespan without nutrients of 12 days, calculated from having no cost of producing TGFβ and no benefit from TGFβ signaling. TN cell division depends directly on bone-derived nutrients through:


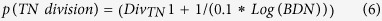


where 0 < BDN < 1 and *Div*_*TN*_ is the maximum rate of TN division. Under TGFβ inhibition, the maximum division rate of TN remains the same.

### Cell Culture and Patient Specimens

Luciferase-expressing PAIII, C42B, and PC3 prostate cancer cell lines were cultured in complete Dulbecco’s Modified medium supplemented with 10% fetal bovine serum^58^,^59^,^60^. All cell lines were periodically tested for mycoplasma (#CUL001B, R&D Systems) and short tandem repeat (STR) verified at the Moffitt Clinical Translational Research Core. De-identified tissue sections of bone metastatic prostate cancer were obtained from the Moffitt tissue archives (MCC 50086).

### *In vivo* experiments

All animal experiments were performed with IACUC approval (R1283) and were conducted in accordance with the guidelines set forth in the *Guidelines for the Care and Use of Laboratory Animals* published by the National Institutes of Health. Pre-Treatment Studies: 6-week old male SCID Beige mice were injected intraperitoneally with either TGFβ inhibitor (1D11; 10 μg/ml; n = 10/group) or isotype control, 13C4 (10 μg/ml; n = 8/group), a kind gift from Scott Lonning and Patrick Finn at Genzyme. Subsequently, luciferase expressing PAIII or C4-2B cell lines (5 × 10^4^ or 1 × 10^5^ respectively in 20 μl of saline) were intratibially injected either one-day or one week after TGFβ pre-treatment^61^,^62^. Contra-lateral limbs were injected with saline and served as a positive control. Mice received TGFβ inhibitor or IgG control injections every three days (PAIII model) or weekly (C4-2B). Bioluminescence was measured longitudinally as a correlate of tumor growth (IVIS™ Perkin Elmer). For Post-treatment studies: mice were inoculated as described (n = 7/group), randomized and treated upon the detection of bioluminescent signal. Mice that showed tumor growth outside of the bone compartment were excluded from all analyses.

A detailed description of cell assays, histological and bone morphology analyses can be found in supplementary methods. Statistical analyses were performed with GraphPad Prism and all graphs display error bars that are SEM.

## Additional Information

**How to cite this article**: Cook, L. M. *et al*. Predictive computational modeling to define effective treatment strategies for bone metastatic prostate cancer. *Sci. Rep*. **6**, 29384; doi: 10.1038/srep29384 (2016).

## Supplementary Material

Supplementary Information

Supplementary Video S1

Supplementary Video S2

Supplementary Video S3

Supplementary Table S1

Supplementary Table S2

Supplementary Table S3

## References

[b1] Frieling J. S., Basanta D. & Lynch C. C. Current and emerging therapies for bone metastatic castration-resistant prostate cancer. Cancer control: journal of the Moffitt Cancer Center 22, 109–120 (2015). 25504285 10.1177/107327481502200114 PMC4673894

[b2] Deng X. . Recent advances in bone-targeted therapies of metastatic prostate cancer. Cancer Treat Rev 40, 730–738, doi: 10.1016/j.ctrv.2014.04.003 (2014). 24767837 PMC4042838

[b3] Ottewell P. D., O’Donnell L. & Holen I. Molecular alterations that drive breast cancer metastasis to bone. BoneKEy reports 4, 643, doi: 10.1038/bonekey.2015.10 (2015). 25848532 PMC4371414

[b4] Guise T. A. . Basic mechanisms responsible for osteolytic and osteoblastic bone metastases. Clin Cancer Res 12, 6213s–6216s, doi: 12/20/6213s (2006). 17062703 10.1158/1078-0432.CCR-06-1007

[b5] Juarez P. & Guise T. A. TGF-beta in cancer and bone: implications for treatment of bone metastases. Bone 48, 23–29, doi: S8756-3282(10)01401-8 (2010). 20699127 10.1016/j.bone.2010.08.004

[b6] Anderson A. R. & Quaranta V. Integrative mathematical oncology. Nat Rev Cancer 8, 227–234, doi: nrc2329 (2008). 18273038 10.1038/nrc2329

[b7] Basanta D., Gatenby R. A. & Anderson A. R. Exploiting evolution to treat drug resistance: combination therapy and the double bind. Molecular pharmaceutics 9, 914–921, doi: 10.1021/mp200458e (2012). 22369188 PMC3325107

[b8] Eikenberry S. E., Nagy J. D. & Kuang Y. The evolutionary impact of androgen levels on prostate cancer in a multi-scale mathematical model. Biology direct 5, 24, doi: 10.1186/1745-6150-5-24 (2010). 20406442 PMC2885348

[b9] Gatenby R. A., Silva A. S., Gillies R. J. & Frieden B. R. Adaptive therapy. Cancer Res 69, 4894–4903, doi: 10.1158/0008-5472.CAN-08-3658 (2009). 19487300 PMC3728826

[b10] Horn M. . Model-based decision rules reduce the risk of molecular relapse after cessation of tyrosine kinase inhibitor therapy in chronic myeloid leukemia. Blood 121, 378–384, doi: 10.1182/blood-2012-07-441956 (2013). 23175686

[b11] Leder K. . Mathematical modeling of PDGF-driven glioblastoma reveals optimized radiation dosing schedules. Cell 156, 603–616, doi: 10.1016/j.cell.2013.12.029 (2014). 24485463 PMC3923371

[b12] Rockne R., Alvord E. C. Jr., Rockhill J. K. & Swanson K. R. A mathematical model for brain tumor response to radiation therapy. J Math Biol 58, 561–578, doi: 10.1007/s00285-008-0219-6 (2009). 18815786 PMC3784027

[b13] Swanson K. R. . Quantifying the role of angiogenesis in malignant progression of gliomas: in silico modeling integrates imaging and histology. Cancer Res 71, 7366–7375, doi: 10.1158/0008-5472.CAN-11-1399 (2011). 21900399 PMC3398690

[b14] Swanson K. R., Rostomily R. C. & Alvord E. C. Jr. A mathematical modelling tool for predicting survival of individual patients following resection of glioblastoma: a proof of principle. British Journal of Cancer 98, 113–119, doi: 10.1038/sj.bjc.6604125 (2008). 18059395 PMC2359692

[b15] Rejniak K. A. & Anderson A. R. Hybrid models of tumor growth. *Wiley interdisciplinary reviews*. Systems biology and medicine 3, 115–125, doi: 10.1002/wsbm.102 (2011). 21064037 PMC3057876

[b16] Anderson A. R., Weaver A. M., Cummings P. T. & Quaranta V. Tumor morphology and phenotypic evolution driven by selective pressure from the microenvironment. Cell 127, 905–915, doi: 10.1016/j.cell.2006.09.042 (2006). 17129778

[b17] Robertson-Tessi M., Gillies R. J., Gatenby R. A. & Anderson A. R. Impact of metabolic heterogeneity on tumor growth, invasion, and treatment outcomes. Cancer Res 75, 1567–1579, doi: 10.1158/0008-5472.CAN-14-1428 (2015). 25878146 PMC4421891

[b18] Araujo A., Cook L. M., Lynch C. C. & Basanta D. An integrated computational model of the bone microenvironment in bone-metastatic prostate cancer. Cancer Res 74, 2391–2401, doi: 10.1158/0008-5472.CAN-13-2652 (2014). 24788098 PMC4023121

[b19] Gallaher J. . Improving Treatment Strategies for Patients with Metastatic Castrate Resistan Prostate Cancer through Personalized Computational Modeling. Clin Exp Metastasis In Press (2014). 10.1007/s10585-014-9674-1 PMC5399888 25173680

[b20] Zhao B., Pritchard J. R., Lauffenburger D. A. & Hemann M. T. Addressing Genetic Tumor Heterogeneity through Computationally Predictive Combination Therapy. Cancer Discov 4, 166–174, doi: 10.1158/2159-8290.CD-13-0465 (2014). 24318931 PMC3975231

[b21] Berges R. R. . Implication of cell kinetic changes during the progression of human prostatic cancer. Clin Cancer Res 1, 473–480 (1995). 9816006 PMC4086477

[b22] Morris J. C. . Phase I study of GC1008 (fresolimumab): a human anti-transforming growth factor-beta (TGFbeta) monoclonal antibody in patients with advanced malignant melanoma or renal cell carcinoma. PLoS One 9, e90353, doi: 10.1371/journal.pone.0090353 (2014). 24618589 PMC3949712

[b23] Korpal M. . Imaging transforming growth factor-beta signaling dynamics and therapeutic response in breast cancer bone metastasis. Nat Med 15, 960–966, doi: 10.1038/nm.1943 (2009). 19597504

[b24] Korpal M. & Kang Y. Targeting the transforming growth factor-beta signalling pathway in metastatic cancer. Eur J Cancer 46, 1232–1240, doi: 10.1016/j.ejca.2010.02.040 (2010). 20307969

[b25] Bierie B. & Moses H. L. Tumour microenvironment: TGFbeta: the molecular Jekyll and Hyde of cancer. Nat Rev Cancer 6, 506–520 (2006). 16794634 10.1038/nrc1926

[b26] Pfeilschifter J. . Chemotactic response of osteoblastlike cells to transforming growth factor beta. J Bone Miner Res 5, 825–830, doi: 10.1002/jbmr.5650050805 (1990). 2239366

[b27] Filvaroff E. . Inhibition of TGF-beta receptor signaling in osteoblasts leads to decreased bone remodeling and increased trabecular bone mass. Development 126, 4267–4279 (1999). 10477295 10.1242/dev.126.19.4267

[b28] Janssens K., Ten Dijke P., Janssens S. & Van Hul W. Transforming growth factor-beta1 to the bone. Endocr.Rev. 26, 743–774 (2005). 15901668 10.1210/er.2004-0001

[b29] Edwards J. R. . Inhibition of TGF-beta signaling by 1D11 antibody treatment increases bone mass and quality *in vivo*. J Bone Miner Res 25, 2419–2426, doi: 10.1002/jbmr.139 (2010). 20499365

[b30] Ganapathy V. . Targeting the Transforming Growth Factor-beta pathway inhibits human basal-like breast cancer metastasis. Mol Cancer 9, 122, doi: 1476-4598-9-122 (2010). 20504320 10.1186/1476-4598-9-122 PMC2890606

[b31] Brown K. A., Pietenpol J. A. & Moses H. L. A tale of two proteins: differential roles and regulation of Smad2 and Smad3 in TGF-beta signaling. J Cell Biochem 101, 9–33, doi: 10.1002/jcb.21255 (2007). 17340614

[b32] Fuller K., Lean J. M., Bayley K. E., Wani M. R. & Chambers T. J. A role for TGFbeta(1) in osteoclast differentiation and survival. J Cell Sci 113 (Pt 13), 2445–2453 (2000). 10852823 10.1242/jcs.113.13.2445

[b33] Hughes D. E. . Estrogen promotes apoptosis of murine osteoclasts mediated by TGF-beta. Nat Med 2, 1132–1136 (1996). 8837613 10.1038/nm1096-1132

[b34] Cook L. M., Shay G., Aruajo A. & Lynch C. C. Integrating new discoveries into the “vicious cycle” paradigm of prostate to bone metastases. Cancer Metastasis Rev, doi: 10.1007/s10555-014-9494-4 (2014). PMC4096318 24414228

[b35] Basanta D. & Anderson A. R. Exploiting ecological principles to better understand cancer progression and treatment. Interface focus 3, 20130020, doi: 10.1098/rsfs.2013.0020 (2013). 24511383 PMC3915838

[b36] Altrock P. M., Liu L. L. & Michor F. The mathematics of cancer: integrating quantitative models. Nat Rev Cancer 15, 730–745, doi: 10.1038/nrc4029 (2015). 26597528

[b37] Benzekry S. . Classical mathematical models for description and prediction of experimental tumor growth. PLoS Comput Biol 10, e1003800, doi: 10.1371/journal.pcbi.1003800 (2014). 25167199 PMC4148196

[b38] Ditlev J. A., Mayer B. J. & Loew L. M. There is more than one way to model an elephant. Experiment-driven modeling of the actin cytoskeleton. Biophysical journal 104, 520–532, doi: 10.1016/j.bpj.2012.12.044 (2013). 23442903 PMC3566448

[b39] Buenzli P. R., Pivonka P., Gardiner B. S. & Smith D. W. Modelling the anabolic response of bone using a cell population model. J Theor Biol 307, 42–52, doi: 10.1016/j.jtbi.2012.04.019 (2012). 22579551

[b40] Eudy R. J., Gastonguay M. R., Baron K. T. & Riggs M. M. Connecting the Dots: Linking Osteocyte Activity and Therapeutic Modulation of Sclerostin by Extending a Multiscale Systems Model. CPT: pharmacometrics & systems pharmacology 4, 527–536, doi: 10.1002/psp4.12013 (2015). 26451332 PMC4592532

[b41] Graham J. M., Ayati B. P., Holstein S. A. & Martin J. A. The role of osteocytes in targeted bone remodeling: a mathematical model. PLoS One 8, e63884, doi: 10.1371/journal.pone.0063884 (2013). 23717504 PMC3661588

[b42] Komarova S. V. . Mathematical model for bone mineralization. Frontiers in cell and developmental biology 3, 51, doi: 10.3389/fcell.2015.00051 (2015). 26347868 PMC4544393

[b43] Ryser M. D., Nigam N. & Komarova S. V. Mathematical modeling of spatio-temporal dynamics of a single bone multicellular unit. Journal of bone and mineral research: the official journal of the American Society for Bone and Mineral Research 24, 860–870, doi: 10.1359/jbmr.081229 (2009). 19063683

[b44] Ji B., Genever P. G., Patton R. J. & Fagan M. J. Mathematical modelling of the pathogenesis of multiple myeloma-induced bone disease. International journal for numerical methods in biomedical engineering 30, 1085–1102, doi: 10.1002/cnm.2645 (2014). 24817420 PMC4282456

[b45] Ryser M. D., Qu Y. & Komarova S. V. Osteoprotegerin in bone metastases: mathematical solution to the puzzle. PLoS Comput Biol 8, e1002703, doi: 10.1371/journal.pcbi.1002703 (2012). 23093918 PMC3475686

[b46] Anderson A. R. A. . Microenvironmental independence associated with tumor progression. Cancer Res 69, 8797–8806, doi: 10.1158/0008-5472.CAN-09-0437 (2009). 19887618 PMC2783510

[b47] Basanta D. . The role of transforming growth factor-beta-mediated tumor-stroma interactions in prostate cancer progression: an integrative approach. Cancer Res 69, 7111–7120, doi: 0008-5472.CAN-08-3957 (2009). 19706777 10.1158/0008-5472.CAN-08-3957 PMC2748342

[b48] Bonewald L. F. & Mundy G. R. Role of transforming growth factor-beta in bone remodeling. Clinical orthopaedics and related research 261–276 (1990). 2403492

[b49] Kaminska B., Wesolowska A. & Danilkiewicz M. TGF beta signalling and its role in tumour pathogenesis. Acta Biochim Pol 52, 329–337 (2005). 15990918

[b50] Fournier P. G. . The TGF-beta Signaling Regulator PMEPA1 Suppresses Prostate Cancer Metastases to Bone. Cancer Cell doi: 10.1016/j.ccell.2015.04.009 (2015). PMC4464909 25982816

[b51] Juarez P. . Halofuginone inhibits the establishment and progression of melanoma bone metastases. Cancer Res 72, 6247–6256, doi: 10.1158/0008-5472.CAN-12-1444 (2012). 23002206 PMC4447239

[b52] Biswas S. . Anti-transforming growth factor ss antibody treatment rescues bone loss and prevents breast cancer metastasis to bone. PLoS One 6, e27090, doi: 10.1371/journal.pone.0027090 (2011). 22096521 PMC3214031

[b53] Khin Z. P. . A preclinical assay for chemosensitivity in multiple myeloma. Cancer Res 74, 56–67, doi: 10.1158/0008-5472.CAN-13-2397 (2014). 24310398 PMC3915502

[b54] Ince T. A. . Characterization of twenty-five ovarian tumour cell lines that phenocopy primary tumours. Nat Commun 6, 7419, doi: 10.1038/ncomms8419 (2015). 26080861 PMC4473807

[b55] Enriquez-Navas P. M. . Exploiting evolutionary principles to prolong tumor control in preclinical models of breast cancer. Sci Transl Med 8, 327ra324, doi: 10.1126/scitranslmed.aad7842 (2016). PMC4962860 26912903

[b56] Shinar D. M. & Rodan G. A. Biphasic effects of transforming growth factor-beta on the production of osteoclast-like cells in mouse bone marrow cultures: the role of prostaglandins in the generation of these cells. Endocrinology 126, 3153–3158, doi: 10.1210/endo-126-6-3153 (1990). 2161750

[b57] Karst M., Gorny G., Galvin R. J. & Oursler M. J. Roles of stromal cell RANKL, OPG, and M-CSF expression in biphasic TGF-beta regulation of osteoclast differentiation. J Cell Physiol 200, 99–106, doi: 10.1002/jcp.20036 (2004). 15137062 PMC4547836

[b58] Hodde J. P., Suckow M. A., Wolter W. R. & Hiles M. C. Small intestinal submucosa does not promote PAIII tumor growth in Lobund-Wistar rats. J Surg Res 120, 189–194, doi: 10.1016/j.jss.2003.10.022(2004) . 15234212

[b59] Lin D. L. . Bone metastatic LNCaP-derivative C4-2B prostate cancer cell line mineralizes *in vitro*. Prostate 47, 212–221, doi: 10.1002/pros.1065 (2001). 11351351

[b60] Joseph J. . Disseminated prostate cancer cells can instruct hematopoietic stem and progenitor cells to regulate bone phenotype. Mol Cancer Res 10, 282–292, doi: 10.1158/1541-7786.MCR-11-0404 (2012). 22241219 PMC3307952

[b61] Thiolloy S. . An osteoblast-derived proteinase controls tumor cell survival via TGF-beta activation in the bone microenvironment. PLoS One 7, e29862, doi: 10.1371/journal.pone.0029862 (2012). 22238668 PMC3251607

[b62] Thiolloy S. . Osteoclast-derived matrix metalloproteinase-7, but not matrix metalloproteinase-9, contributes to tumor-induced osteolysis. Cancer Res 69, 6747–6755, doi: 69/16/6747(2009). 19679556 10.1158/0008-5472.CAN-08-3949 PMC2745595

