# Supplementary material for: Predictive computational modeling to define effective treatment strategies for bone metastatic prostate cancer
Source: Sci Rep. 2016 Jul 14;6:29384. doi: 10.1038/srep29384 (PMC4944130; doi:10.1038/srep29384)
Supplement: Supplementary Information [file srep29384-s1.pdf]

SUPPLEMENTARY METHODS, TABLES, FIGURES AND VIDEO LEGENDS FOR: Predictive computational modeling to define effective treatment strategies for bone metastatic prostate cancer.

Leah M. Cook[1*], Arturo Araujo[2*], Julio M. Pow-Sang[3], Mikalai M. Budzevich[4], David Basanta[2#] and Conor C. Lynch[1#]

Authors' Affiliation. [1]Tumor Biology Dept., [2]Integrated Mathematical Oncology Dept., [3]Genitourinary Oncology Dept. and Dept. Cancer Imaging and Metabolism,

Moffitt Cancer Center and Research Institute, Tampa, Florida.

*Co-First Author

[#]Co-Senior Author

Supplementary Methods

Cell assays. *Smad reporter assay*-Prostate cancer cell lines were transduced with the SMAD luciferase reporter and tested according to manufacturer's instructions (CCS-017L, Qiagen). *Colony Formation Assay*-Sterile 3% agarose (#BP164, Fisher) was mixed with 2% DMEM and either, TGFβ inhibitor (1D11; 10μg/ml) or isotype control, (13C4; 10 μ g/ml). PAIII-Luc cells were plated at a density of $5x10^3$ cells/well in soft agar containing α-MEM, 5% fetal bovine serum and 0.7% agarose. Media containing TGFβ inhibitor or IgG control was replenished every other day. After 21 days colonies were stained with crystal violet and the numbers and diameter measured and analyzed using Image J. *Real-time qPCR*-RNA was isolated and reverse transcribed (#4368814, Applied Biosystems). cDNA was amplified with the following mouse/rat primers: TβRI: Forward: 5'-CGCTCTGTCCACGGCAAG-3', Reverse: 5'-TCATGTCTCACAGCAAGTCCC-3', TβRII:Forward:5'-GGCCAAGCTGAAGCAGAAC-3', Reverse: 5'-GGATGTTCTCGTGTTTCAGGTT-3', in addition to human primers TβRI: Forward: 5'-CGAGTGCCAAATGAAGAGGA-3', Reverse: 5'-CGACCTTTGCCAATGCTTTC -3', TβRII: Forward: 5'-ACTTTATTCTGGAAGATGCTGCT-3', Reverse: 5'-GCTGATGCCTGTCACTTGAA-3.' Multi-species 18S was used to normalize values: Forward: 5'-GTAACCCGTTGAACCCCATT-3' Reverse: 5'-CCATCCAATCGGTAGTAGCG-3.' *ELISA*- TGFβ was then measured by ELISA as per manufacturer's instructions (#MB100B, R&D Systems). *Proliferation Assay*-MC3T3-E1 osteoblast precursor cells were serum-starved for 24 hours and treated with increasing concentrations of recombinant TGFβ (#240-B, R&D Systems; 0-50ng/ml) in serum-free alpha-MEM for 24 hours. Changes in proliferation was measured using CellTiter96 Non-Radioactive Proliferation Assay (#G400, Promega), according to protocol.

Microcomputed tomography (μCT) and X-ray. For gross analysis of trabecular bone volume, formalin fixed tibiae were scanned at an isotropic voxel size of 18

μm (Siemens Inveon PET/CT/SPECT scanner with cone beam X-Ray source). The scans were performed at 80kVp voltage and 500 mA current with an exposition time of 3000 ms per projection. Measurements were made as previously described [1]. Radiographic images (Faxitron X-ray Corp) were obtained using an energy of 35kVp and an exposure time of 8 ms. The spatial resolution is 10 lp/mm (48μm). The tumor volume (TuV) was calculated as a function of the total tissue volume (TV) of the tibial medullary canal using ImageJ software.

Histology. De-identified human bone metastases tissue sections were obtained via the Moffitt Cancer Center Total Cancer Care protocol. Specimens were rehydrated and blocked prior to the addition of specific primary antibody (pan-Cytokeratin, #C2562, Sigma-Aldrich; TGFβRII, #sc-400, Santa Cruz; TGFβ1 #MAB240, R&D Systems; Cleaved Caspase-3, #9964S, Cell Signaling; phospho-Histone H3, #04-1093, Millipore; pSMAD2, #AB3849, Millipore; SMAD2, #sc-6033 Santa Cruz, pAKT, #sc-16646, Santa Cruz; AKT, #4691, Cell Signaling) and appropriate IgG controls. Subsequently, species-specific secondary AlexaFluor 568 and AlexaFluor 488-conjugated antibodies (1: 1000 dilution for one hour at room temperature; #A10042 and #A21202 Invitrogen) were added for imaging by microscopy. For semi-quantitative analysis, regional images were segmented based on the intensity of staining using Definions Tissue Studio (TS)©.

Osteoclast and osteoblast measurement. Tartrate-resistant acid phosphatase (TRAcP) was detected according to the manufacturer's instructions (Kit 387-A, Sigma-Aldrich). For histological quantitation of bone volume and osteoblast numbers, mouse tibia were additionally stained with Gomori's Trichrome stain [2]. Sections were scanned and quantified using Spectrum analysis software and Image J.

Statistical Analysis. Statistical analyses were performed using GraphPad Prism (GraphPad Software, Inc).

Table S1. Parameters (empirically derived or assumed) used partial differential equations. ***Ts*** refers to time steps, ***Px*** refers to 10µm^2, ***a*** refers to assumed data, ***b***, assumed from the diameter of an osteoclast and the amount of bone resorbed over a 24-hour period, ***c***, empirical data based on PAIII survival in low serum soft agar assays. A penalty is applied to each clone based on their production of the ligand and receptor (+/- 4 days).

Parameter	Value	Normalized value	Ref.
**OBL Diameter**	15µm	1 px	[3]
**OCL Diameter**	50-100µm	3-5px	[4]
**OBL Speed**	10µm/hr	1/10px/ts	[5,6]
**OCL Speed**	100µm/hr	1px/ts	[5,7,8]
**OCL TGFβ Production**	0.00558ng/day	1 MaxTGFβ/ts	a
**Cancer TGFβ Production**	0.005pg/day	0.0001 MaxTGFβ/ts	a
**Rate of bone degradation**	10µm/day	1/240px/ts	[9,10]
**Resorption Pit Area**	7.58x 10^{-14} m^3	70px	b
**Rate of one formation**	0.656 µm/day	1/850px/ts	[10,11]
**BMU size**	2 x 0.5 mm^2	200x50px	a
**TGFβ diffusion**	750 μm^2/min	0.01px/ts	[12-14]
**TGFβ half-life**	2 min	0.5ts	[15,16]
**TR Survival**	10 days +/- 4	2400 +/- 960 ts	c
**TRP Survival**	10 days +/- 4	2400 +/- 960 ts	c
**TP Survival**	10 days	2400 ts	c
**TN Survival**	10 days	2400 ts	c
**TR Proliferation**	1.75 days	420 ts	c
**TRP Proliferation**	1.5 days	360 ts	c
**TP Proliferation**	1.75 days	420 ts	c
**TN Proliferation**	1.25 days	300 ts	c

Supplementary Table Figure Legends

Note: Supplementary Table S2, S3 and, S4 are uploaded as individual excel spreadsheets.

Table S2. The effect of varying levels of TGFβ inhibitor efficacy on cell populations during normal bone remodeling. Data points are captured from Day 20 and Day 79. MSC= mesenchymal stromal cell, pOB= precursor osteoblast, aOB = adult osteoblast, pOC = precursor osteoclast, aOC = adult osteoclast.

Table S3. TGFβ inhibition of the PCa-BME was tested at varying doses, from 0-100% and applied at Day 80 (Post-treatment) or Day 1 (Pre-treatment) of the 250 day simulation. Numbers represent average cell number or bone (in μm) of 25 simulations at Day 100, at the peak of tumor growth rate with standard deviation, and at Day 250, at the end of simulation. PCa= prostate cancer, MSC= mesenchymal stromal cell, pOB= precursor osteoblast, aOB = adult osteoblast, pOC = precursor osteoclast, aOC = adult osteoclast.

Table S4. TGFβ inhibition of the PCa-BME was applied at Day 1 of the 250 day simulation (Pre-treatment), prior to tumor seeding, to a heterogeneous population of TRP (TGFB receptor and ligand producing), TR (receptor-expressing), and TN (negative for TGFB receptors and ligands). Numbers represent average cell number or bone (in μm) of 25 simulations at Day 100 and at Day 250 (end of simulation), at the peak of tumor growth rate with standard deviation. TR = PCa= prostate cancer, MSC= mesenchymal stromal cell, pOB= precursor osteoblast, aOB = adult osteoblast, pOC = precursor osteoclast, aOC = adult osteoclast.

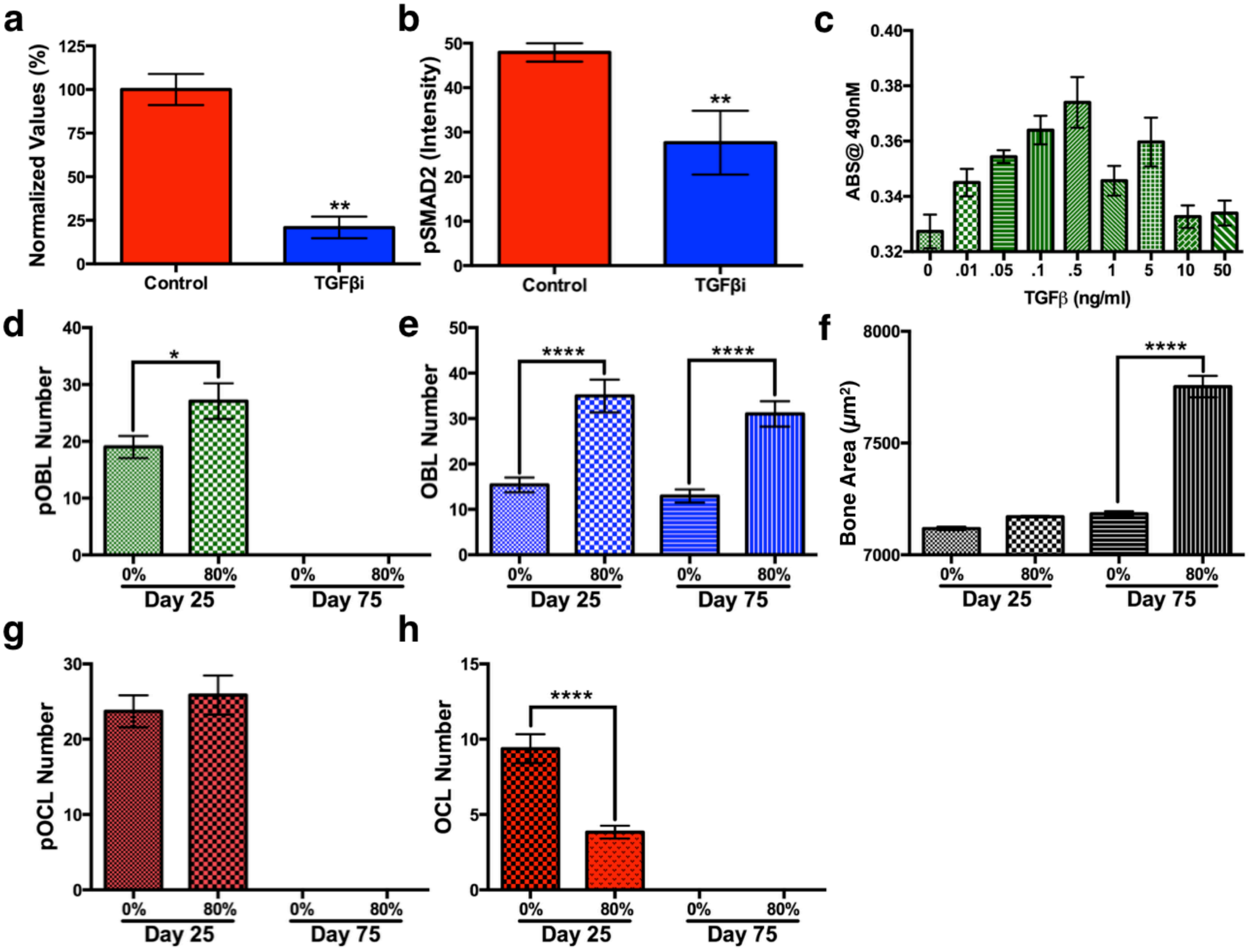

Figure S1. TGFβ inhibition stimulates bone formation during normal bone remodeling. **(a, b)** Tumor naïve mice were treated with TGFβ inhibitor (1D11, 10mg/Kg) or isotype control (13C4, 10mg/Kg) and circulating levels of TGFβ were measured in serum by ELISA and normalized to total protein concentration (a). The level of pSMAD2 positivity in bones derived from control and TGFβ inhibitor treated animals (b) was also determined by measuring pSMAD2 intensity with Definiens Tissue Studio. **(c)** Proliferation, as a measure of absorbance, of MC3T3-E1, precursor osteoblast cells, treated with increasing concentrations of recombinant TGFβ for 24 hours. **(d-h)** In silico analysis of TGFβ inhibition (applied at 80% efficacy) in the normal bone modeling unit on

precursor (d) and mature (e) osteoblast numbers, total bone area (f), and precursor (g) and mature (h) osteoclast numbers over a 75-day period. Graphs represent the numbers of cell populations in control and TGFβ groups at the day 25 and day 75 time points. Asterisks denote the following p-values; *, $p<0.05$; ** $p<0.01$, ****, $p<0.001$.

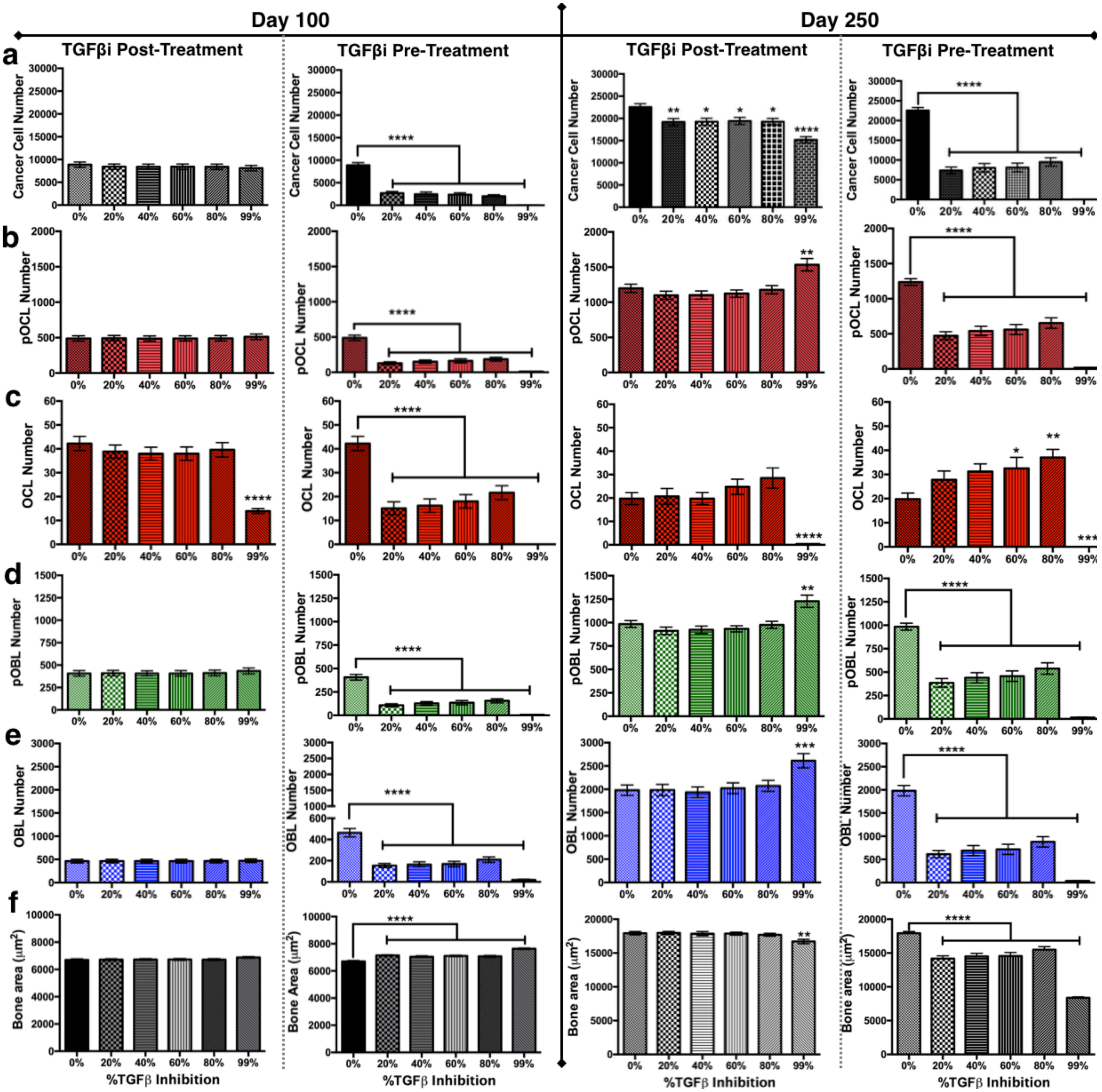

Figure S2. Comparative *in silico* analyses of TGFβ treatment with varying levels of efficacy (0-99%) on metastatic prostate cancer-bone microenvironment cell populations at days 100 and 250. The inhibitor was applied *in silico* to metastases that were already established (post-treatment) or prior to the inoculation of the model with metastatic prostate cancer cells (pre-treatment).

Results were calculated at day 100 and 250. **(a)** *In silico* analysis of prostate cancer cell numbers in post and pre-treatment groups. **(b,c)** The impact of TGFβ inhibition on osteoclast precursor (b) and mature osteoclast (c) numbers. **(d-f)** The numbers of osteoblast precursors (d), bone forming osteoblasts (e) and the changes in bone area (f) in response to TGFβ inhibition were also determined in post- and pre-treatment settings. Asterisks denote statistical significance (*, $p<0.05$; **, $p<0.005$; ***, $p<0.001$; ****, $p<0.0001$).

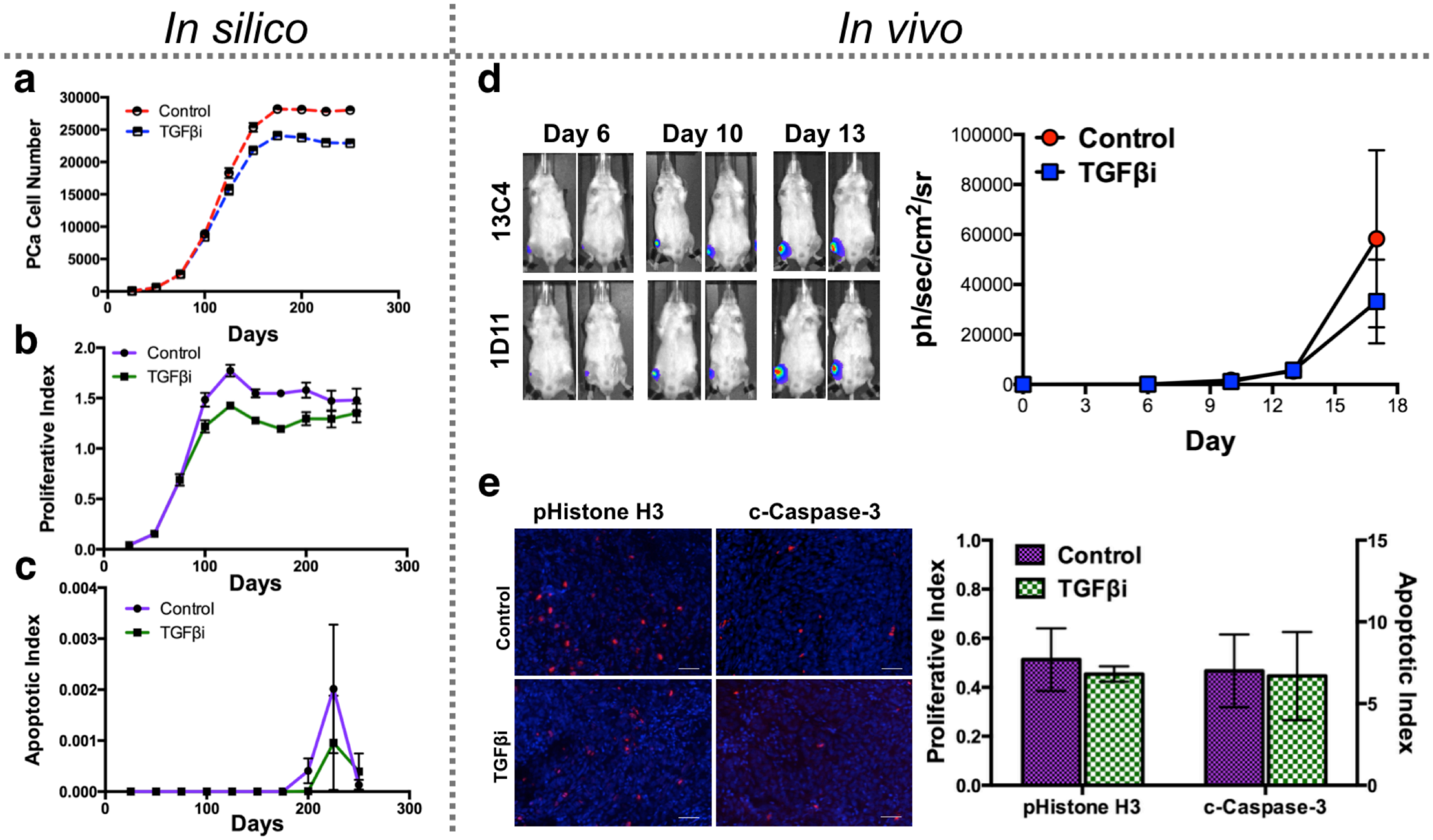

Figure S3. Validation of TGFβi post-treatment *in silico* predictions. **(a-c)** In computational model simulations, TGFβ inhibition was applied to bone metastatic lesions at day 80. The impact on cancer cell growth (a), proliferative index (b) and, apoptotic index (c). **(d)** The effect of TGFβi (1D11, 10µg/ml; n=7) versus control (13C4, 10µg/ml; n=7) was determined on established PAIII intratibial lesions in vivo using bioluminescence. Mice were treated twice weekly after randomization on day 6 post tumor inoculation. **(e)** The proliferative and apoptotic indices in TGFβi and control tissue sections were measured using pHistone H3 and cleaved caspase-3 (c-Caspase-3) positivity (red) respectively as a ratio to total cell number (DAPI; blue).

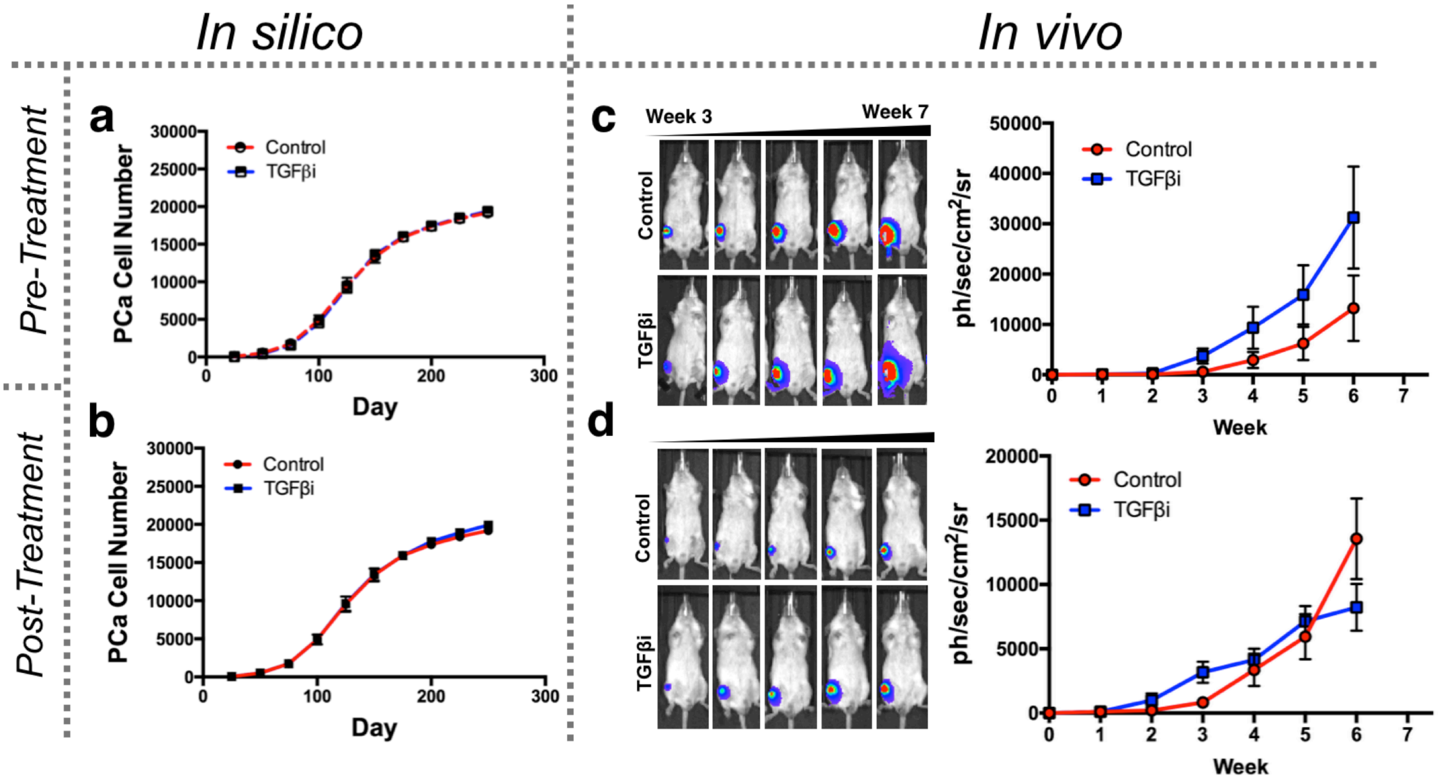

Figure S4. The *in silico* and *in vivo* effects of TGFβ inhibition on TGFβ producing (TP) cells. **(a-b)** In computational model simulations, TGFβ inhibition (at a level of 80% efficacy) was applied in a pre- or post-treatment manner and the effect on tumor growth simulated (n≥25). For pre-treatment conditions (a), inhibition was applied at the start of the simulation and for post-treatment conditions (b), inhibition was applied at Day 80; **(c-d)** To validate computational model results regarding the impact of TGFβ inhibition on a TP population, we used C4-2B cells. C4-2B luciferase expressing cells were intratibially inoculated into control mice or mice pre- (c) or post-treated (d) with TGFβ inhibitor. Representative images of bioluminescence for each group are shown.

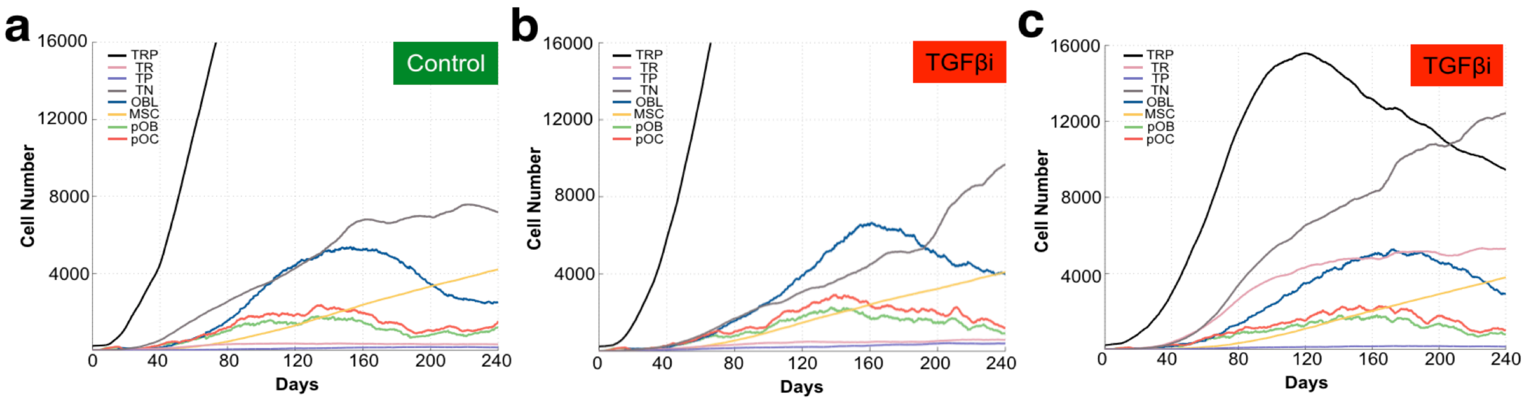

Figure S5. Analysis of TRP, TR, TP, TN and bone microenvironmental population dynamics over time. **(a-c)**. The computational model was seeded with patient derived information regarding the ratio of TGFβ utilizing clones (TP 1: TRP 231: TR 6: TN 4). Simulations (≥24) were then performed under control (a) or TGFβ inhibition (80% efficacy) at post- (inhibition applied at Day 80) and pre-treatment conditions (inhibition applied at the start of the simulation) (b, c). Blue shading identifies initiation of post-treatment with TGFβ inhibitor. Graphs are representative of a single computational model output.

Video S1. Impact of TGFβ inhibition on established bone metastatic prostate cancer growth (post-treatment). Metastases (TRP) were seeded into the HCA model and allowed to grow for 80 days prior to the application of TGFβ inhibition at a level of 80% efficacy. Upper left quartile illustrates the dynamic changes in cancer and bone microenvironment populations over time (250 days). Clockwise, other quartiles show TGFβ, bone derived nutrient and RANKL availability (hotter colors indicate higher concentrations of each factor). HCA Control simulation is shown on left with TGFβ inhibitor simulation on right. Individual cells are identified as: mesenchymal stromal cells (gold), osteoblasts (blue), osteoclasts/precursor osteoclasts (red), precursor osteoblasts (green), and TRP cancer cells (black).

Video S2. Impact of TGFβ inhibition prior to the seeding of metastatic prostate cancer cells. TGFβ inhibition (at a level of 80% efficacy) was applied to the HCA model prior to the *in silico* inoculation of prostate cancer cells (TRP). Upper left quartile illustrates the dynamic changes in cancer and bone microenvironment populations over time (250 days). Clockwise, other quartiles show TGFβ, bone derived nutrient and RANKL availability (hotter colors indicate higher concentrations of each factor). Control simulation is shown on left with TGFβ inhibitor simulation on right. Individual cells are identified as: mesenchymal stromal cells (gold), osteoblasts (blue), osteoclasts/precursor osteoclasts (red), precursor osteoblasts (green), and TRP cancer cells (black).

Video S3. TGFβ inhibition effects on TRP, TR, TP and TN population dynamics. TGFβ inhibition (at a level of 80% efficacy) was applied to the HCA model prior to the *in silico* inoculation of TRP (black), TR (pink), TP (purple) and TN (grey) clones. Upper left quartile illustrates the dynamic changes in cancer and bone microenvironment populations over time (250 days). Clockwise, other quartiles show TGFβ, bone derived nutrient and RANKL availability (hotter colors indicate higher concentrations of each factor). Control simulation is shown on left with TGFβ inhibitor simulation on right. Individual cells are identified as: mesenchymal

stromal cells (gold), osteoblasts (blue), osteoclasts/precursor osteoclasts (red), and precursor osteoblasts (green).

Supplementary References

1 Thiolloy, S. *et al.* An osteoblast-derived proteinase controls tumor cell survival via TGF-beta activation in the bone microenvironment. *PLoS One* **7**, e29862, doi:10.1371/journal.pone.0029862(2012).

2 Halpern, J. *et al.* The application of a murine bone bioreactor as a model of tumor: bone interaction. *Clin Exp Metastasis* **23**, 345-356 (2006).

3 Jayakumar, P. & Di Silvio, L. Osteoblasts in bone tissue engineering. *Proc Inst Mech Eng H* **224**, 1415-1440 (2010).

4 Roodman, G. D. Osteoclast differentiation. *Crit Rev Oral Biol Med* **2**, 389-409 (1991).

5 Ferrier, J., Xia, S. L., Lagan, E., Aubin, J. E. & Heersche, J. N. Displacement and translocation of osteoblast-like cells by osteoclasts. *J Bone Miner Res* **9**, 1397-1405, doi:10.1002/jbmr.5650090911 (1994).

6 Shin, H., Zygourakis, K., Farach-Carson, M. C., Yaszemski, M. J. & Mikos, A. G. Attachment, proliferation, and migration of marrow stromal osteoblasts cultured on biomimetic hydrogels modified with an osteopontin-derived peptide. *Biomaterials* **25**, 895-906 (2004).

7 Dacquin, R. *et al.* Control of bone resorption by semaphorin 4D is dependent on ovarian function. *PLoS One* **6**, e26627, doi:10.1371/journal.pone.0026627 (2011).

8 Monchau, F. *et al.* In vitro studies of human and rat osteoclast activity on hydroxyapatite, beta-tricalcium phosphate, calcium carbonate. *Biomol Eng* **19**, 143-152 (2002).

9 Wergedal, J., Stauffer, M., Baylink, D. & Rich, C. Inhibition of bone matrix formation, mineralization, and resorption in thyroparathyroidectomized rats. *J Clin Invest* **52**, 1052-1058, doi:10.1172/JCI107270 (1973).

10 Kanehisa, J. & Heersche, J. N. Osteoclastic bone resorption: in vitro analysis of the rate of resorption and migration of individual osteoclasts. *Bone* **9**, 73-79 (1988).

11 Bloebaum, R. D., Bachus, K. N., Momberger, N. G. & Hofmann, A. A. Mineral apposition rates of human cancellous bone at the interface of porous coated implants. *J Biomed Mater Res* **28**, 537-544, doi:10.1002/jbm.820280503 (1994).

12 UBC. *Diffusion rates for molecules* <http://www.math.ubc.ca/~ais/website/status/diffuse.html>

13 Basanta, D. *et al.* The role of transforming growth factor-beta-mediated tumor-stroma interactions in prostate cancer progression: an integrative approach. *Cancer Res* **69**, 7111-7120, doi:0008-5472.CAN-08-3957(2009).

14 Christley, S., Alber, M. S. & Newman, S. A. Patterns of mesenchymal condensation in a multiscale, discrete stochastic model. *PLoS Comput Biol* **3**, e76, doi:10.1371/journal.pcbi.0030076 (2007).

15 Kaminska, B., Wesolowska, A. & Danilkiewicz, M. TGF beta signalling and its role in tumour pathogenesis. *Acta Biochim Pol* **52**, 329-337 (2005).

16 Wakefield, L. M. *et al.* Recombinant latent transforming growth factor beta 1 has a longer plasma half-life in rats than active transforming growth

factor beta 1, and a different tissue distribution. *J Clin Invest* **86**, 1976-1984, doi:10.1172/JCI114932 (1990).
